# Discriminant validity and interrelations of conceptual and procedural knowledge in fractions and algebra: Evidence from confirmatory factor analysis

**DOI:** 10.1111/bjep.70091

**Published:** 2026-05-11

**Authors:** Michael D'Erchie, Claire L. Forsmann, Michael Schneider, Andreas Obersteiner

**Affiliations:** ^1^ Department of Educational Sciences Technical University of Munich, Heinz Nixdorf Chair of Mathematics Education Munich Germany; ^2^ Department of Educational Psychology Trier University Trier Germany

**Keywords:** algebra, conceptual and procedural knowledge, confirmatory factor analysis, discriminant validity, fractions

## Abstract

**Background:**

Conceptual and procedural knowledge are two distinct types of mathematical knowledge. Measuring them with sufficient discriminant validity is challenging because they are typically highly correlated. Prior studies have demonstrated discriminant validity of paper‐and‐pencil measures separately for fractions and algebra. However, whether such measures can distinguish between the two knowledge types within a single sample of students instructed in both domains, and how the four constructs interrelate, remains unclear.

**Aims:**

This study tested the discriminant validity of conceptual and procedural knowledge measures of fractions and algebra in a single sample of students instructed in both domains and examined the full pattern of intercorrelations to clarify which knowledge types underlie the well‐established fraction–algebra link.

**Sample:**

Participants were 571 German middle‐school students (*M* = 14.91 years).

**Methods:**

Conceptual and procedural knowledge in both domains was assessed using adapted paper‐and‐pencil tests. Confirmatory factor analysis was used to evaluate the discriminant validity of the measures and to examine the intercorrelations among the four constructs.

**Results:**

A four‐factor model showed the best fit, indicating empirical separability of the four constructs. Conceptual and procedural knowledge were highly correlated within each domain (fractions and algebra). Descriptively, cross‐domain correlations were stronger for corresponding knowledge types (e.g., conceptual‐conceptual) than for non‐corresponding types (e.g., conceptual‐procedural), and stronger for the conceptual‐conceptual than the procedural‐procedural link.

**Conclusion:**

The findings contribute to discussions about the discriminant validity and measurability of conceptual and procedural knowledge and offer a more differentiated view of how specific types of knowledge link fractions and algebra.

## INTRODUCTION

### The distinction between conceptual and procedural knowledge

Research in educational psychology and mathematics education has long distinguished between conceptual and procedural knowledge (for reviews, see Castro et al., 2016; Rittle‐Johnson, 2019; Rittle‐Johnson & Siegler, 1998). *Conceptual knowledge* refers to the comprehension of mathematical ideas, principles and their interrelationships within a particular domain (e.g., Hiebert & Lefevre, 1986; Rittle‐Johnson et al., 2001). It is typically characterized as flexible, general and abstract—the kind of knowledge that enables a student to explain why a mathematical procedure works, to judge whether an answer is reasonable, to recognize connections between different representations of the same concept, or to transfer understanding to novel problem situations (e.g., Rittle‐Johnson, 2019). In contrast, a *procedure* is a “step‐by‐step instruction that prescribes how to complete a task” (Hiebert & Lefevre, 1986, p. 6). Procedural knowledge refers to knowledge of rules, procedures and algorithms used to solve problems (Byrnes & Wasik, 1991; Hiebert & Lefevre, 1986). It is usually assumed to be less flexible and more closely tied to specific problem types, contexts and practiced routines and is typically assessed through students' ability to execute procedures (e.g., Rittle‐Johnson, 2019).

### Importance and challenges of measuring conceptual and procedural knowledge with discriminant validity

To understand relationships between conceptual and procedural knowledge, it is fundamental to have measures that assess both knowledge types with sufficient discriminant validity (e.g., Rittle‐Johnson, 2019; Schneider & Stern, 2010). *Discriminant validity* is a psychometric concept rooted in construct validity theory (Campbell & Fiske, 1959). It refers to the extent to which measures of distinct theoretical constructs are also empirically distinguishable—that is, whether they capture unique variance rather than measuring essentially the same thing. Being able to measure conceptual and procedural knowledge with sufficient discriminant validity is important. For research, independent measurement is a prerequisite for studying how these knowledge types relate, develop over time, or respond to instructional interventions. For instance, if a teaching method targets conceptual understanding, its impact can only be assessed if conceptual knowledge can be measured with sufficient discriminant validity; otherwise, effects may be confounded with changes in procedural knowledge. For educational practice, separate measures allow educators to identify which type of knowledge a student lacks and to tailor instruction accordingly (e.g., emphasizing sense‐making for students with weak conceptual knowledge, or guided practice for those lacking procedural knowledge).

Despite this importance, creating measures with sufficient discriminant validity is difficult. One reason is that the two knowledge types tend to be highly correlated (e.g., Hallett et al., 2010; Hecht et al., 2003; Jordan et al., 2013; Schneider et al., 2011). This frequently observed high correlation may be a consequence of instruction—the two knowledge types are often taught and practiced in close temporal proximity—and their developmental relationship. According to the *iterative model*, conceptual and procedural knowledge grow in tandem through mutually reinforcing cycles (e.g., Rittle‐Johnson et al., 2001). For example, a student who initially learns to solve equations by mechanically performing the same operation on both sides may, through repeated practice, come to understand why this procedure preserves equality. This conceptual insight, in turn, enables the student to flexibly adapt the procedure to unfamiliar equation types, such as equations with variables on both sides. Over time, this cycle increasingly entangles the two knowledge types, which is desirable from a learning perspective but creates a psychometric challenge. Despite this challenge, researchers have worked to develop paper‐and‐pencil measures that distinguish between the two knowledge types (e.g., Lenz et al., 2020). Paper‐and‐pencil tests are attractive because they can be administered to large samples, unlike more resource‐intensive alternatives, such as think‐aloud protocols, clinical interviews, or process‐tracing methods, that are less practical for large samples.

### Evidence in support of conceptual and procedural knowledge measures' discriminant validity

One commonly used way to provide evidence in support of measures' discriminant validity is through structural equation modelling, and confirmatory factor analysis in particular. Structural equation modelling enables the modelling of unobservable constructs—such as conceptual and procedural knowledge—as latent variables. These latent variables capture the variance shared among their observable indicators (so‐called manifest variables) and thus represent the underlying construct these indicators are assumed to measure (e.g., Brown, 2015). For example, procedural knowledge can be modelled as a latent variable inferred from multiple manifest indicators, such as items assessing fraction arithmetic. One way to examine discriminant validity is to specify conceptual and procedural knowledge as separate but correlated latent factors and to compare this two‐factor model with a more parsimonious one‐factor model that treats all items as indicators of a single undifferentiated latent construct. If the two‐factor model fits the data significantly better, this provides evidence that the measures capture distinct constructs (e.g., Rönkkö & Cho, 2022).

Following this approach, two studies have provided evidence that paper‐and‐pencil measures can distinguish conceptual and procedural knowledge in mathematics with sufficient discriminant validity. Lenz et al. (2020) examined fraction knowledge in German 8th‐ and 9th‐graders. Conceptual knowledge was assessed using translation tasks (between visual and symbolic representations), number line tasks and explanation tasks targeting students' understanding of fraction concepts, while procedural knowledge was assessed using calculation tasks (e.g., fraction arithmetic, expanding and simplifying fractions) and procedure‐verbalization tasks. A two‐factor CFA model fit the data significantly better than a one‐factor model, supporting their measures' discriminant validity. Their results also revealed which items best captured each construct: number line tasks and part‐whole understanding tasks (e.g., shading fractional parts of a polygon) were the strongest indicators of conceptual knowledge, while fraction addition/subtraction and fraction conversion items (e.g., $\frac{14}{5}=2\frac{4}{5}$) best indicated procedural knowledge.

Similarly, Schneider et al. (2011) studied algebra knowledge in two U.S. samples of 7th‐ and 8th‐graders. Conceptual knowledge was assessed using items targeting students' understanding of algebraic concepts such as equivalence and the meaning of variables (e.g., judging and explaining whether expressions are equivalent), whereas procedural knowledge was assessed through equation‐solving tasks. Schneider and colleagues found that a model with two interrelated latent factors—one for conceptual knowledge and the other for procedural knowledge—fit the data well. Together, these two studies provide evidence that conceptual and procedural knowledge can be empirically distinguished within individual mathematical domains using paper‐and‐pencil measures.

### Discriminant validity of conceptual and procedural knowledge in a joint cross‐domain model

The two studies reviewed above demonstrated discriminant validity within individual domains. An open question is whether typical measures of conceptual and procedural knowledge show sufficient discriminant validity in a single sample of students who have been instructed in both domains. There are at least two reasons why this cannot simply be assumed. First, addressing this question requires testing a four‐factor model that distinguishes not only between conceptual and procedural knowledge within each domain but also between knowledge types across domains. With more latent factors sharing variance, the risk of empirical indistinguishability increases. This is because some mathematical concepts and procedures span both domains—for example, the concept of equivalence is relevant to both simplifying fractions and solving equations, and symbolic manipulation is involved in both fraction arithmetic and algebraic transformation—which may make it harder to cleanly separate the four constructs empirically. Second, the iterative model predicts that conceptual and procedural knowledge become increasingly intertwined with instruction (Rittle‐Johnson et al., 2001). Because fractions are typically taught before algebra, students who have completed instruction in both domains have had more time for the iterative cycle to operate than students tested shortly after fraction instruction alone. This may be further accelerated by algebra instruction, which frequently requires students to apply fraction skills and thus provides additional opportunities to integrate conceptual and procedural fraction knowledge. For algebra, Schneider et al. (2011) found that latent factor intercorrelations between conceptual and procedural algebra knowledge were lower prior to instruction but substantially higher afterward, and interpreted this finding as increased knowledge integration. Therefore, the degree of measures' discriminant validity may depend on the characteristics of the student population being assessed—most importantly, on the amount of instructional experience students have had with the relevant content.

At the same time, addressing this question is important. On the one hand, a joint model yields factor loadings that reveal which item types most effectively capture each knowledge type, which can inform future test development and the design of assessments that maximize discriminant validity. This is particularly relevant for algebra, where Schneider et al. (2011) used item parcelling that blended different content areas into composite indicators, leaving it unclear which specific content areas best capture conceptual and procedural algebra knowledge. Because we constructed separate indicators for distinct algebra content areas (see the Measures section), our approach can address this limitation. We will return to this point in the Discussion section. More importantly, however, testing discriminant validity is a psychometric prerequisite for examining how conceptual and procedural knowledge relate across fractions and algebra—a question whose theoretical and practical significance is discussed in the next section. If the four constructs cannot be empirically separated using paper‐and‐pencil measures, examining their interrelations would not be meaningful.

### Relationships between knowledge of fractions and algebra

A large body of research has established that students' knowledge of fractions is substantially related to their knowledge of algebra (e.g., Booth & Newton, 2012; Ching & Li, 2024; DeWolf et al., 2015; Hurst & Cordes, 2018; Liang et al., 2018; Powell et al., 2019; Silla et al., 2024; Viegut et al., 2024). This relationship has been found consistently across countries, age groups and study designs, and it persists even after controlling for general intelligence, working memory, reading comprehension, whole‐number arithmetic and socioeconomic background (Siegler et al., 2012). The fraction–algebra link is therefore one of the most robust findings in recent research on mathematical development. Yet, despite this robust overall association, its nature is not obvious. After all, fractions are part of the number domain and require arithmetic skills. Algebra, on the other hand, requires working with abstract symbols rather than specific numbers. It is therefore not immediately clear how and why knowledge of fractions should transfer to knowledge of algebra. A first approach to clarifying this relationship is to examine whether the link is primarily driven by conceptual knowledge, procedural knowledge, or both. If conceptual and procedural knowledge in fractions and algebra can be measured with sufficient discriminant validity in a sample of students instructed in both domains, this would allow us to address this gap by examining the full correlation structure among conceptual fraction knowledge, procedural fraction knowledge, conceptual algebra knowledge and procedural algebra knowledge.

Conceptual and procedural knowledge of fractions have been conceptualized in prior work (e.g., Lenz et al., 2020). Conceptual fraction knowledge involves understanding of various interpretations of fractions (for an overview, see Kieren, 1976), such as fractions as representations of part‐whole relationships or as numbers with measurable magnitudes that can be compared and ordered on a number line. It also includes understanding how properties of fractions differ from natural or whole numbers (for an overview, see Obersteiner et al., 2016). For example, unlike integers, there is always another fraction between any two fractions (density property). Conceptualizations of procedural fraction knowledge focus on fraction arithmetic and comprise the ability to perform fraction addition, subtraction, multiplication and division as well as operations like expanding or simplifying fractions (e.g., $\frac{18}{24}=\frac{6}{8}=\frac{3}{4}$).

In contrast to fractions, algebra is a broad‐ranging field of mathematics. In the present study, we focus on linear equations because they are typically the first algebra topic students encounter after fractions (Staatsinstitut für Schulqualität und Bildungsforschung, n.d.) and because prior research on the relationship between fraction and algebra knowledge has focused on this topic (e.g., Barbieri et al., 2021; Booth et al., 2014; Booth & Newton, 2012; DeWolf et al., 2015; Hurst & Cordes, 2018), allowing us to connect our research to previous work.

Regarding algebra, and equation solving specifically, several authors have described relevant facets of conceptual and procedural knowledge (e.g., Knuth et al., 2011; Rittle‐Johnson et al., 2009; Schneider et al., 2011). Conceptual algebra knowledge encompasses a rich understanding of at least three core aspects: variables, equivalence and equations. Understanding variables involves recognizing variables as symbols representing unknown or varying quantities and understanding that they can be manipulated within algebraic expressions and equations (e.g., Asquith et al., 2007; Knuth et al., 2011). Algebraic equivalence refers to the idea that different expressions can represent the same value, as well as knowing that performing identical operations on both sides of an equation maintains its equality. Conceptual understanding of equations includes knowing that equations are used to describe equalities and model real‐life situations and are not just statements to solve, as well as knowing efficient steps to solve a given equation (e.g., Alibali et al., 2014). Procedural algebra knowledge involves the ability to systematically manipulate equations symbolically by transforming algebraic expressions, combining like terms and applying operations such as addition, subtraction, multiplication and division on both sides of an equation to isolate variables.

Knowing how these two knowledge types (conceptual and procedural) relate across domains (fractions and algebra) is important. From a theoretical perspective, it can clarify which specific knowledge types underlie the well‐established fraction–algebra link. For instance, it is reasonable to expect within‐type cross‐domain correlations (e.g., conceptual – conceptual) to be stronger than cross‐type cross‐domain correlations (e.g., conceptual – procedural) because conceptual knowledge across domains may draw on similar forms of reasoning such as relational thinking, while procedural knowledge may rely on shared skills such as symbolic fluency (e.g., Viegut, 2021). If this pattern holds, it could point to potential underlying cognitive mechanisms that can then be tested in subsequent studies and may help to better understand *why* fraction knowledge relates to algebra knowledge. More generally, examining the full correlation structure also contributes to discussions about the interplay between conceptual and procedural knowledge both within and across domains. For example, it allows us to compare how tightly conceptual and procedural knowledge are related within each domain. The iterative model posits a positive within‐domain association between the two knowledge types but does not specify whether this association should be stronger in some domains than others. There are reasons to expect a stronger coupling in algebra, where conceptual understanding—such as grasping the principle of equivalence—appears particularly directly relevant to procedural skill in equation solving. In fractions, by contrast, students often execute procedures with insufficient conceptual understanding (e.g., Kerslake, 1986; Siegler & Lortie‐Forgues, 2015), which would imply a weaker coupling. Yet the opposite prediction is also defensible: because fractions are typically learned earlier, the iterative cycle may have had more time to tighten the conceptual–procedural link in that domain. Comparing the within‐domain correlations for fractions and algebra within the same sample and at the same point in time allows this question to be addressed empirically for the first time. The full correlation structure also allows us to examine whether within‐domain correlations uniformly exceed cross‐domain correlations. This might be expected given that fractions and algebra are distinct mathematical domains, taught in separate instructional units at different points in time. However, this expectation has not been tested empirically.

From an educational practice perspective, quantifying the correlations is also important. Current classroom teaching of fractions tends to emphasize procedural knowledge, so students often show a poorly developed conceptual understanding of fractions (e.g., Bempeni et al., 2018; Lortie‐Forgues et al., 2015; Vamvakoussi & Vosniadou, 2010). If, for example, the relationship between fractions and algebra is predominantly explained by correlations between conceptual knowledge, this may suggest that conceptual fraction knowledge is particularly important for later conceptual algebra learning, and that placing greater emphasis on conceptual understanding during fraction instruction could better prepare students for later algebra.

### The present study

To summarize, conceptual and procedural knowledge are widely recognized as two distinct types of mathematical knowledge whose separate measurement is important for theory, instruction, or intervention research. However, as argued above, measuring them with sufficient discriminant validity is challenging as both knowledge types are often closely intertwined. Two prior studies (e.g., Lenz et al., 2020; Schneider et al., 2011) have demonstrated discriminant validity within individual domains—one for fractions, one for algebra—but it remains unclear whether paper‐and‐pencil measures of conceptual and procedural knowledge in both fractions and algebra show discriminant validity in a single sample of students instructed in both domains. The latter is a psychometric prerequisite to investigate how conceptual and procedural knowledge interrelate across fractions and algebra—a question still unclear, despite the well‐established overall association between fractions and algebra.

The present study addressed these two gaps. We used and adapted previously validated test instruments for conceptual and procedural knowledge in both fractions and algebra and administered them as paper‐and‐pencil tests to a sample of German middle‐school students with a mean age of approximately 15 years, all of whom had completed school instruction in both domains. If discriminant validity is supported, we further examine the full pattern of intercorrelations among conceptual fraction knowledge, procedural fraction knowledge, conceptual algebra knowledge and procedural algebra knowledge and test whether these correlations differ in strength. This corresponds to the following two research questions:

1. Do paper‐and‐pencil measures of conceptual and procedural knowledge in fractions and algebra demonstrate sufficient discriminant validity in students who have completed instruction in both domains?
2. If so, how are conceptual fraction knowledge, procedural fraction knowledge, conceptual algebra knowledge, and procedural algebra knowledge correlated, and do these correlations differ in strength?

## METHODS

### Sample

Participants were 571 students (248 girls, 294 boys, 11 gender diverse, 18 students did not provide data on their gender; *M*_age_ = 14.91 years, *SD* = 0.74 years) from 26 classes across five academic‐track secondary schools in Germany. Data were collected in 2023. In the German school system, fractions are typically introduced in Grade 6, while algebra—including variables, equivalence and linear equation solving—is introduced in Grade 7. Both topics occur frequently in subsequent grades. Our participants attended Grades 9 and 10 and can thus be considered experienced in both domains. Students' prior mathematics achievement was about average based on their self‐reported end‐of‐year mathematics grade in the previous school year (*M* = 2.87, *SD* = 1.05; on a scale from 1 = very good to 6 = insufficient). Self‐report was necessary because data protection regulations prohibited schools from sharing student records with the research team. Of the 571 participants, 536 completed the fractions test and 494 completed the algebra test, with missing data attributable to illness or other incidental absences.

### Procedure

Prior to data collection, the study was approved by the Ethics Committee of Trier University (16/2020), the Bavarian Ministry of Education, Germany (Reference: IV.7‐BO5106/246/11), and the responsible local education authority in Rhineland‐Palatinate, Germany (Reference: 553‐22). Schools and teachers were informed about the study in advance and agreed to participate. Informed parental permission and student assent were obtained from all participants prior to the data collection. Data were collected in whole‐class settings for two 45‐min sessions. Brief general instructions were given at the beginning of each lesson. Participants worked on the fraction test in the first lesson and on the algebra test in the second. The first author, the second author, or a trained research assistant administered the tests.

### Measures

#### General design principles and adaptations of existing test instruments

For both the fractions and algebra test instruments, we measured conceptual and procedural knowledge using three manifest indicators each (described below). Three indicators per knowledge type were selected because this is the minimum number required for the measurement model to be overidentified (i.e., *df* >0), thereby enabling evaluation of model fit.

Conceptual knowledge is typically assessed using a broad range of task formats that emphasize understanding without requiring computation: translating between different representations, comparing quantities, evaluating whether mathematical statements or examples are conceptually valid, or generating and selecting definitions of key concepts, whereas procedural knowledge is usually assessed more uniformly by presenting students with familiar types of problems that can be solved by executing a known or taught algorithm, with performance scored on the accuracy of answers or solution steps (for an overview, see Rittle‐Johnson, 2019). We used these types of tasks to assess the two knowledge types. We assessed both knowledge types using adapted versions of the instruments by Lenz et al. (2020) for fractions and Schneider et al. (2011) for algebra. Adaptations were made to suit our target population, to broaden construct coverage and to ensure balanced measurement models.

For fractions, we included more challenging items to avoid ceiling effects, as Lenz and colleagues had sampled younger students from the intermediate Realschule track, whereas we sampled students from the Gymnasium track (the highest academic track in the German school system, preparing students for university entrance). Regarding conceptual knowledge, Lenz and colleagues included two separate indicators focusing on fraction magnitude understanding, which we consolidated into a single indicator. To cover the construct more broadly—in line with recommendations in the literature (Rittle‐Johnson, 2019)—we added a new indicator on students' conceptual understanding of the density property of fractions (i.e., between any two fractions there are infinitely many other fractions). For procedural knowledge, their instrument covered addition and subtraction but omitted multiplication and division. We therefore added a dedicated indicator for fraction multiplication and division. As a third procedural indicator, we retained their expanding and simplifying indicator.

For algebra, Schneider et al. (2011) assessed conceptual knowledge through items covering equivalence and variable understanding. However, because they aggregated items into composite indicators (through odd/even item parcelling) to form manifest variables, it remains unclear which of these content dimensions most strongly drives the latent construct as the parcelling procedure blends both content dimensions into each indicator rather than separating them. To better inform future test design, we instead constructed manifest variables that map onto distinct content dimensions: variable, equivalence and equation understanding, with the latter added as a novel third indicator to reach the desired number of three indicators per knowledge type. We also broadened the operationalization of procedural knowledge beyond solving multi‐step linear equations by adding indicators for equations with parameters and for setting up equations from word problems.

To ensure that each manifest variable was measured with an equal number of items to produce balanced measurement models, we supplemented our item pool with additional items sourced from other published studies (Knuth et al., 2011; McMullen & Van Hoof, 2020; Otten et al., 2019; Reinhold, 2018; Rittle‐Johnson & Star, 2007; Rosnick, 1981; Sleeman, 1984; Vamvakoussi & Vosniadou, 2010) where necessary. The resulting instruments are described in detail below, with example items given in Table 1.

**TABLE 1:** Example Items for Conceptual and Procedural Knowledge About Fractions and Algebra.

Measure	Example item
*Conceptual fraction knowledge*	
Part‐Whole Relations Understanding (FC1)	The sketch shows $\frac{2}{3}$ of a figure. 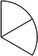 Draw the whole figure.
Magnitude Understanding (FC2)	Tom wants to know which of the two fractions $\frac{8}{9}$ and $\frac{7}{6}$ is larger (without calculating/expanding). Mark the larger fraction and briefly explain your decision.
Density Understanding (FC3)	How many fractions lie between $\frac{3}{8}$ and $\frac{7}{8}$? Three Five A finite number of fractions, but more than five Infinitely many
*Procedural fraction knowledge*	
Addition and Subtraction (FP1)	Calculate: $\frac{10}{11}-\frac{9}{10}$
Multiplication and Division (FP2)	Calculate: $\frac{5}{7}\cdot \frac{3}{7}$
Expanding and Simplifying (FP3)	Simplify the fraction $\frac{28}{42}$ as much as possible.
*Conceptual algebra knowledge*	
Variable Understanding (AC1)	The letter x represents any number. Which of the expressions 3x and x+6 has a greater value? Mark each statement as true or false. 3x is always greater than x+6. x+6 is always greater than 3x. 3x is always equal to x+6. The question cannot be answered because necessary information is missing. The question cannot be answered as the two expressions contradict each other.
Equivalence Understanding (AC2)	Are the two equations: 2x+15=31 and –2x–15=–31 equivalent to each other? Briefly explain.
Equation Understanding (AC3)	Maria and Erika are supposed to solve the equation 7·(x–2)=3·(x–2)+16. Below you can see the solution paths of Maria and Erika. 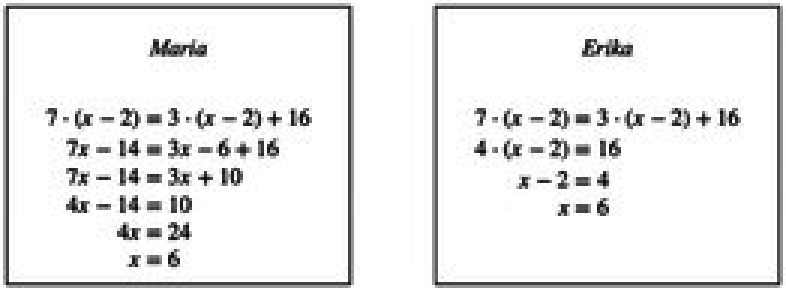 Maria and Erika have used different solution paths yet arrived at the same (correct) solution. Explain how Erika proceeded so that her solution path is shorter than Maria's.
*Procedural algebra knowledge*	
Solving Equations (AP1)	Solve for x: 5x–1–x–1=–2x–6+3x–2
Solving Equations with Parameters (AP2)	Solve for x: 3a=4x+1
Setting up and Solving Equations (AP3)	In Christian's terrarium, there are four beetles (6 legs) and spiders (8 legs). In total, all the animals in the terrarium have 120 legs. How many spiders are there in the terrarium? Write an equation to describe this situation. Use x as a variable for the number of spiders and solve the equation.

*Note*: FC1−FC3, FP1−FP3 were assessed with 5 items each. AC1−AC3, AP1−AP3 were assessed with four items each.

#### Test instrument on conceptual and procedural fraction knowledge

Students' fraction knowledge was measured with a total of 30 items (Cronbach's α = .78). Out of these 30 items, 15 aimed to measure conceptual fraction knowledge (α = .67) and the remaining 15 items aimed to measure procedural knowledge (α = .73). None of the items contained any unknowns or algebraic variables. For conceptual fraction knowledge, the three indicators assessed were understanding *fractions as representations of part‐whole relationships*, *fraction magnitude* and *fraction density*. All manifest variables were operationalized by a sum score computed over five items.

Items that measured students' understanding of fractions as representations of part‐whole relationships included problems where students were asked to shade parts of objects indicated by a fraction symbol or to select the fraction that correctly represents the area of a shaded polygon. For fraction magnitude, students had to locate a specific fraction on a number line, identify a fraction marked on a number line, or compare two fraction magnitudes using brief non‐computational explanation. To measure students' understanding of the density property of fractions, we included items that required students to decide how many fractions are between two given fractions or to explain whether it is possible to give a fraction between two given fractions.

For procedural fraction knowledge, the facets were *fraction addition and subtraction*, *fraction multiplication and division*, and *expanding and simplifying fractions*. Again, each of these three facets were operationalized as a sum score over five items. Fraction addition and subtraction items required students to solve problems involving addition and subtraction of fractions. Numerators and denominators were integers ranging from 1 to 11. There were no pairs of fractions with the same denominator, so students had to first find a common denominator, extend both fractions to the common denominator, and then add/subtract the resulting numerators. Fraction multiplication and division items involved tasks requiring students to multiply and divide fractions. Numerators and denominators were integers ranging from 1 to 10. Items on expanding and simplifying fractions required students to expand a fraction to an equivalent form or simplify a fraction to its simplest form.

#### Test instrument on conceptual and procedural algebra knowledge

The algebra scale comprised a total of 24 items^1^ (α = .78). Twelve items focused on measuring conceptual algebra knowledge (α = .58) and the remaining 12 items focused on procedural algebra knowledge (α = .71). None of the items contained any fractions. We measured three central facets of algebra knowledge: *understanding variables*, *understanding equivalence* and *understanding equations*. Each of these facets was operationalized by a sum score computed over four items.

Conceptual understanding of variables was measured with items requiring students to explain what a variable is, items that required students to understand that variables can stand for unknown numbers and items that tested students' understanding that variables can cover a range of numbers simultaneously. Conceptual equivalence understanding was measured using items in which a pair of equations were given, and students had to indicate and explain whether the equations were equivalent or not or items that asked students to identify equivalent expressions for a given expression. Conceptual understanding of equations and equation‐solving included items requiring students to compare solution paths of multi‐step linear equations, for example, by explaining why a given solution path was more efficient than other paths.

For procedural algebra knowledge, we measured students' ability to solve multi‐step linear equations with and without parameters, as well as setting up and solving a linear equation based on simple word problems. Again, each of the procedures was operationalized by a sum score computed over four items. In all items for procedural algebra knowledge, the unknown x could appear on both sides of the equation or only on one side. The coefficients in the equations were all integers.

### Coding

Items were coded dichotomously (1 = correct; 0 = incorrect). Some open response items on the conceptual knowledge scales were coded using a three‐level coding scale (1 = correct; 0.5 = partially correct; 0 = incorrect) to account for partially correct responses. The first author coded the tests. A total of 31% of the participants' fraction and algebra tests were randomly selected and double‐coded by a trained research assistant (for all items: Cohen's κ ≥ .85). In cases of disagreement, the first author revisited the respective items and discussed the coding decisions with the research assistant based on the coding manual until consensus was reached. The proportion of cases requiring discussion was very small (1.26% of all cases), and no systematic pattern of disagreement was observed. The impact of these cases on the results is therefore considered negligible.

### Data analyses

To address the first research question, we followed a three‐step procedure. First, we examined whether fraction knowledge and algebra knowledge demonstrate discriminant validity. To this end, we compared a two‐factor model, specifying separate but correlated latent factors for fraction and algebra knowledge, with a one‐factor model representing general mathematical knowledge. We expected the two‐factor model to provide a better fit, as the algebra items did not involve fractions.

Second, we investigated, separately for fractions and algebra, whether conceptual and procedural knowledge can be empirically distinguished. For each domain, we specified a two‐factor model (with correlated latent factors for conceptual and procedural knowledge) and compared it to a one‐factor model. Based on prior findings (Lenz et al., 2020; Schneider et al., 2011), we expected the two‐factor models to fit the data better.

Finally, we specified a four‐factor model comprising separate but intercorrelated latent factors for conceptual fraction knowledge, procedural fraction knowledge, conceptual algebra knowledge and procedural algebra knowledge. This model allowed us to examine the discriminant validity as well as the intercorrelations of all four constructs simultaneously.

Following best practices in structural equation modelling (e.g., Cheung et al., 2024; Rönkkö & Cho, 2022; Strauss & Smith, 2009), we also compared the final four‐factor model with more parsimonious alternatives. These alternative models included (a) a one‐factor model assuming that mathematical performance is best represented by a unidimensional factor, with no distinction between domains (fractions vs. algebra) or type of knowledge (conceptual vs. procedural); (b) a two‐factor model only distinguishing between fraction and algebra knowledge; (c) a two‐factor model only distinguishing between conceptual and procedural knowledge; (d) a three‐factor model differentiating between conceptual and procedural knowledge within algebra but treating fraction knowledge as undifferentiated; (e) a three‐factor model differentiating between conceptual and procedural knowledge within fractions but treating algebra knowledge as undifferentiated; (f) a three‐factor model with domain‐general conceptual knowledge and domain‐specific procedural knowledge; and (g) a three‐factor model with domain‐general procedural knowledge and domain‐specific conceptual knowledge. Table 2 gives an overview of all tested models^2^ including their latent and manifest variables. Manifest variables are presented in Table 1.

**TABLE 2:** Overview of All Models Estimated.

Model tested	Latent variables	Manifest variables
*Step 1: Separability of fraction and algebra knowledge*		
2‐factor model	Fraction knowledge Algebra knowledge	FC1–FC3, FP1–FP3 AC1–AC3, AP1–AP3
1‐factor model	Math knowledge	FC1–FC3, FP1–FP3, AC1–AC3, AP1–AP3
*Step 2: Within‐domain separability of conceptual and procedural knowledge*		
Fractions		
2‐factor model	Conceptual fraction knowledge Procedural fraction knowledge	FC1–FC3 FP1–FP3
1‐factor model	Fraction knowledge	FC1–FC3, FP1–FP3
Algebra		
2‐factor model	Conceptual algebra knowledge Procedural algebra knowledge	AC1–AC3 AP1–AP3
1‐factor model	Algebra knowledge	AC1–AC3, AP1–AP3
*Step 3: Cross‐domain separability of conceptual and procedural fraction and algebra knowledge*		
4‐factor model (hypothesized model)	Conceptual fraction knowledge Procedural fraction knowledge Conceptual algebra knowledge Procedural algebra knowledge	FC1–FC3 FP1–FP3 AC1–AC3 AP1–AP3
*Alternative models*		
1‐factor model	Math knowledge	FC1–FC3, FP1–FP3, AC1–AC3, AP1–AP3
2‐factor model	Fraction knowledge Algebra knowledge	FC1–FC3, FP1–FP3 AC1–AC3, AP1–AP3
2‐factor model	Conceptual knowledge Procedural knowledge	FC1–FC3, AC1–AC3 FP1–FP3, AP1–AP3
3‐factor model	Fraction knowledge Conceptual algebra knowledge Procedural algebra knowledge	FC1–FC3, FP1–FP3 AC1–AC3 AP1–AP3
3‐factor model	Conceptual fraction knowledge Procedural fraction knowledge Algebra knowledge	FC1–FC3 FP1–FP3 AC1–AC3, AP1–AP3
3‐factor model	Conceptual knowledge Procedural fraction knowledge Procedural algebra knowledge	FC1–FC3, AC1–AC3 FP1–FP3 AP1–AP3
3‐factor model	Conceptual fraction knowledge Conceptual algebra knowledge Procedural knowledge	FC1–FC3 AC1–AC3 FP1–FP3, AP1–AP3

*Note*: Abbreviations for manifest variables as in Table 1.

It is important to note that we did not assume any of the alternative models to be theoretically more plausible than the four‐factor model. Rather, testing these alternative models had methodological reasons and is consistent with best practices in structural equation modelling, which recommend evaluating more constrained models with collapsed latent factors to assess discriminant validity and model adequacy (e.g., Brown, 2015; Kline, 2023). Simply demonstrating that the hypothesized four‐factor model fits the data well may leave open the question of whether only a theoretically preferred model was evaluated. By systematically comparing the four‐factor model with more parsimonious alternatives, we aimed to provide stronger evidence that the data genuinely support the proposed structure and that no simpler model offers a better representation of the data.

To address the second research question, contingent on the four‐factor model providing the best fit to the data, we conducted a series of model comparisons using the hypothesized four‐factor model as reference. In each comparison, we tested a modified version of this model in which a pair of inter‐factor correlations is constrained to be equal. We then compared the fit of each constrained model to that of the unconstrained four‐factor model using χ^2^ – difference tests. A significant χ^2^ – difference test indicates that the constraint leads to a worse model fit, suggesting that the two correlations significantly differ in magnitude.

All data analyses were performed in *R* (R version 4.5.1; R Core Team, 2025), primarily using the *lavaan*‐package (package version 0.6.19; Rosseel, 2012) for structural equation modelling and confirmatory factor analyses. Missing values were addressed using the Full Information Maximum Likelihood (FIML) estimation method as implemented in the *lavaan* package. For model identification, the loading of the first indicator for each factor was fixed to one. To address non‐normal distributions, we used robust maximum‐likelihood estimation with robust standard errors (White, 1980) and scaled test statistics (asymptotically equal to Yuan‐Bentler scaled test statistics; Yuan & Bentler, 1998). We accounted for the nesting of students within classrooms in all models tested using the *cluster* function of the lavaan package.

Model fit was evaluated based on the Comparative Fit Index (CFI) and Tucker‐Lewis Index (TLI), with values greater than 0.95 reflecting a good fit. The Root Mean Square Error of Approximation (RMSEA) and Standardized Root Mean Square Residual (SRMR) were also assessed, with RMSEA values below 0.05 and SRMR values below 0.08 considered a good fit. Model comparisons were further informed by the Akaike Information Criterion (AIC), Bayesian Information Criterion (BIC) and sample‐size adjusted BIC (sBIC), for which lower values suggest a better fitting model. Additionally, χ^2^ – difference tests were used to compare models.

## RESULTS

### Descriptives

Table 3 shows the descriptive statistics and intercorrelations for the 12 indicator variables of fraction and algebra knowledge. The mean percentages of accurate responses were in the mid‐range of the scale (*M* = 56–86%). All measures had substantial individual differences (*SD* ≥ 17%). The indicator variables showed statistically significant correlations, ranging from low to moderate (.12 ≤ *r* ≤ .52).

**TABLE 3:** Descriptive Statistics and Correlation Matrix of the Manifest Measures.

	*M*	*SD*	Skewness	Kurtosis	(1)	(2)	(3)	(4)	(5)	(6)	(7)	(8)	(9)	(10)	(11)
*Fractions*															
Part‐Whole Relations Understanding (1)	.86	.17	−1.30	1.62	‐										
Magnitude Understanding (2)	.72	.24	−0.65	−0.22	.36	‐									
Density Understanding (3)	.61	.30	−0.30	−1.15	.27	.43	‐								
Addition and Subtraction (4)	.84	.25	−1.96	3.49	.26	.32	.28	‐							
Multiplication and Division (5)	.78	.29	−1.28	0.74	.12	.21	.21	.31	‐						
Expanding and Simplifying (6)	.73	.28	−0.72	−0.37	.22	.26	.25	.31	.29	‐					
*Algebra*															
Variable Understanding (7)	.63	.24	−0.28	−0.52	.24	.31	.31	.22	.16	.22	‐				
Equivalence Understanding (8)	.58	.30	−0.34	−0.81	.31	.34	.45	.33	.28	.32	.37	‐			
Equation Understanding (9)	.68	.21	−0.07	−0.41	.21	.29	.28	.25	.16	.28	.31	.39	‐		
Solving Equations (10)	.65	.32	−0.43	−0.96	.16	.30	.27	.35	.27	.29	.21	.38	.36	‐	
Solving Equations With Parameters (11)	.56	.34	−0.22	−1.17	.28	.31	.33	.31	.34	.28	.31	.44	.32	.52	‐
Setting up and Solving Equations (12)	.68	.24	−0.57	−0.33	.27	.31	.36	.33	.21	.29	.34	.43	.34	.35	.40

*Note*: All correlations are statistically significant at *p* < .01.

### Separability of fraction and algebra knowledge

As described in the Data analysis section, we first compared a two‐factor model (with latent factors for *fraction* and *algebra knowledge*) with a one‐factor model (with a single latent factor for *mathematical knowledge*). The results are summarized in Table 4.

**TABLE 4:** Model Fit Indices Comparing the Two‐Factor (Fractions and Algebra) and the One‐Factor (Math Knowledge) Model.

Model description	χ^2^	*df*	CFI	TLI	RMSEA	SRMR	AIC	BIC	sBIC
2‐factor model (latent factors for *fraction knowledge* and *algebra knowledge*)	110.6***	53	.950	.938	.051	.038	17,990	18,151	18,034
1‐factor model (one factor for *mathematical knowledge*)	119.5***	54	.943	.930	.055	.040	18,001	18,158	18,043

***p < .001.

The two‐factor model showed a better relative model fit than the one‐factor model in terms of having higher CFI and TLI values and lower RMSEA, SRMR, AIC, BIC and sBIC values. The two factors were intercorrelated (*r* = .90, 95% CI [.83, .98], *p* < .001). Standardized factor loadings ranged from .43 to .70 (all *p*s < .001) for the two‐factor model and .42 to .69 (all *p*s < .001) for the one‐factor model. The difference in model fit between the two models was statistically significant, χ^2^(1) = 5.45, *p* < .05. This indicates that knowledge of fractions and knowledge of algebra are partly empirically distinguishable knowledge types.

### Within‐domain separability of conceptual and procedural knowledge

Next, separately for the domains of fractions and algebra, we compared a two‐factor model (distinguishing between conceptual and procedural knowledge) with a one‐factor model (not distinguishing between conceptual and procedural knowledge). The results are summarized in Table 5.

**TABLE 5:** Model Fit Indices Comparing Two‐Factor (Conceptual vs. Procedural) and One‐Factor Models for the Domains of Fractions and Algebra.

Model description	χ^2^	*df*	CFI	TLI	RMSEA	SRMR	AIC	BIC	sBIC
*Fractions*									
2‐factor model (latent factors for *conceptual fraction knowledge* and *procedural fraction knowledge*)	6.48	8	1.00	1.00	.000	.018	10,115	10,197	10,136
1‐factor model (one factor for *fraction knowledge*)	30.80***	9	.942	.904	.072	.038	10,141	10,218	10,161
*Algebra*									
2‐factor model (latent factors for *conceptual algebra knowledge* and *procedural* *algebra knowledge*)	24.80**	8	.971	.946	.070	.030	8107	8187	8127
1‐factor model (one factor for *algebra knowledge*)	30.13***	9	.960	.934	.077	.033	8114	8189	8132

***p < .001, **p < .01.

For fractions, the two‐factor model showed a good absolute, and a better relative model fit than the one‐factor model in terms of having higher CFI and TLI values and lower RMSEA, SRMR, AIC, BIC and sBIC values. Conceptual and procedural fraction knowledge were correlated (*r* = .72, 95% CI [.61, .83], *p* < .001). Standardized factor loadings ranged from .49 to .70 (all *p*s < .001) in the two‐factor model and from .41 to .65 (all *p*s < .001) in the one‐factor model. The difference in model fit was significant, χ^2^(1) = 30.45, *p* < .001. This indicates that conceptual and procedural fraction knowledge were partly distinguishable.

For algebra, the two‐factor model showed an acceptable‐to‐good absolute fit and exhibited a slightly better fit than the one‐factor model, with marginally higher CFI and TLI values and slightly lower RMSEA, SRMR, AIC, BIC and sBIC values. The two latent factors were highly correlated (*r* = .88, 95% CI [.78, .98], *p* < .001). Standardized factor loadings for the two‐factor model ranged from .52 to .71 (all *p*s < .001), and for the one‐factor model from .49 to .68 (all *p*s < .001). The difference in model fit was significant (χ^2^ (1) = 4.56, *p* < .05), suggesting partial empirical separability between conceptual and procedural algebra knowledge.

### Cross‐domain separability of conceptual and procedural fraction and algebra knowledge

To investigate the cross‐domain separability of conceptual and procedural fraction and algebra knowledge, we evaluated a model with four intercorrelated latent factors representing conceptual knowledge of fractions, procedural knowledge of fractions, conceptual knowledge of algebra and procedural knowledge of algebra. Goodness‐of‐fit indices indicated that the four‐factor model fit the data well (see Table 6).

**TABLE 6:** Model Fit Indices for the Four‐Factor Model and All Tested Alternative Models.

Model description	χ^2^	*df*	CFI	TLI	RMSEA	SRMR	AIC	BIC	sBIC
*Hypothesized* *Model*									
4‐factor model (latent factors for *conceptual, procedural fraction knowledge* and *conceptual* and *procedural algebra knowledge*)	71.5*	48	.979	.971	.036	.031	17,956	18,138	18,005
*Alternative* *Models*									
1‐factor model (one factor for *general mathematical knowledge*)	119.5***	54	.943	.930	.055	.040	18,001	18,158	18,043
2‐factor model (latent factors for *fraction knowledge* and *algebra knowledge*)	110.6***	53	.950	.938	.051	.038	17,990	18,151	18,034
2‐factor model (latent factors for *conceptual knowledge* and *procedural knowledge*)	96.1***	53	.962	.953	.045	.036	17,975	18,136	18,018
3‐factor model (latent factors for *fraction knowledge, conceptual and procedural algebra knowledge*)	102.8***	51	.956	.944	.049	.037	17,984	18,154	18,030
3‐factor model (latent factors for *conceptual, procedural fraction knowledge* and *algebra knowledge*)	85.7**	51	.968	.958	.042	.034	17,966	18,136	18,012
3‐factor model (latent factors for *conceptual knowledge, procedural fraction knowledge* and *procedural algebra knowledge*)	85.0**	51	.970	.961	.041	.034	17,966	18,135	18,012
3‐factor model (latent factors for *conceptual fraction knowledge, conceptual algebra knowledge* and *procedural knowledge*)	84.2**	51	.970	.962	.040	.033	17,964	18,134	18,010

*p < .05, **p < .01, ***p < .001.

We compared the four‐factor model to the alternative models as described in the Data analysis section. As shown in Table 6, the four‐factor model had the highest CFI and TLI values, the lowest RMSEA and SRMR values, and the lowest AIC and sample‐size adjusted BIC (sBIC), leading to the conclusion that the four‐factor model fitted the data best. This conclusion was supported by a series of χ^2^ – difference tests, with the four‐factor model showing significantly better model fits compared to all alternative models (all *p*s < .01).

### Correlative relationships between conceptual and procedural fraction and algebra knowledge

**FIGURE 1:**
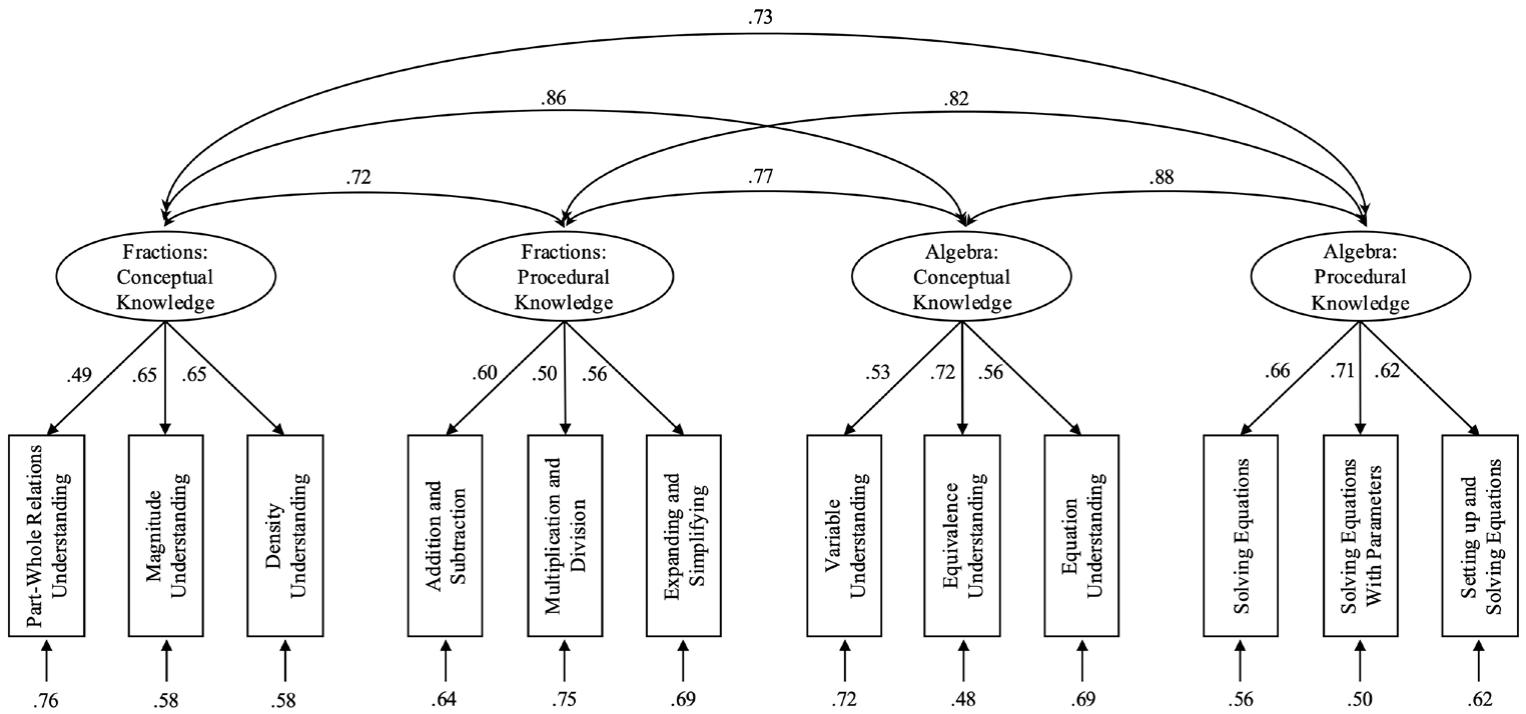
Standardized Factor Loadings and Standardized Covariances for the Four‐Factor Model.

Standardized factor loadings for the best‐fitting four‐factor model ranged from .49 to .72 (all *p*s < .001; see Figure 1). Factor correlations were high between all factors and ranged from .72 to .88 (all *p*s < .001). The highest correlation was found between conceptual and procedural algebra knowledge (*r* = .88, 95% CI [.78, .98]) and the lowest between conceptual and procedural fraction knowledge (*r* = .72, 95% CI [.60, .84]). Descriptively, conceptual fraction knowledge correlated more strongly with conceptual algebra knowledge (*r* = .86, 95% CI [.76, .96]) than with procedural algebra knowledge (*r* = .73, 95% CI [.60, .86]), and procedural fraction knowledge correlated more strongly with procedural algebra knowledge (*r* = .82, 95% CI [.73, .92]) than with conceptual algebra knowledge (*r* = .77, 95% CI [.66, .87]).

To test whether these descriptive differences were statistically meaningful, we compared the relative strength of the correlation coefficients by constraining pairs of correlations to be equal and comparing each constrained model to the unconstrained four‐factor model. However, none of the 15 pairwise comparisons reached statistical significance (all Bonferroni‐corrected *p*s > .29). Therefore, the differences in the strengths of correlations between knowledge facets should be interpreted with caution.

## DISCUSSION

The present study had two aims: to examine whether paper‐and‐pencil measures of conceptual and procedural knowledge in fractions and algebra show sufficient discriminant validity in students after being instructed in both domains, and—if so—to provide a fine‐grained account of which specific knowledge types underlie the well‐established association between students' knowledge of fractions and algebra.

### Discriminant validity of measures of conceptual and procedural knowledge

Our first research question asked whether paper‐and‐pencil measures of conceptual and procedural knowledge in fractions and algebra demonstrate sufficient discriminant validity in students who have completed instruction in both domains. Using confirmatory factor analyses, we found that a four‐factor model—with separate latent factors for conceptual fraction knowledge, procedural fraction knowledge, conceptual algebra knowledge and procedural algebra knowledge—showed a significantly better model fit than all alternative models, as evidenced by the chi‐square difference tests. While chi‐square difference tests can be criticized for being sample size sensitive, the four‐factor model was also preferred across all global fit indices, providing converging support for its best fit. This finding replicates and extends prior work that demonstrated discriminant validity within single domains—fractions in German 8th‐ and 9th‐graders (Lenz et al., 2020) and algebra in American 7th‐ and 8th‐graders shortly after algebra instruction (Schneider et al., 2011)—to a joint cross‐domain model in a sample of German ninth‐ and tenth‐grade middle‐school students who had completed instruction in both domains.

Latent factor correlations were uniformly high (*r* = .72–.88). These high factor correlations may raise questions about the distinctiveness of constructs. However, empirical separability, and relatedly discriminant validity, is not an all‐or‐nothing property but exists along a continuum. Following current guidelines, concerns about insufficient discriminant validity only become serious when confidence intervals for factor correlations include 1 (Rönkkö & Cho, 2022). Importantly, in our data, none of the 95% confidence intervals for the six factor correlations included this upper bound of 1. This suggests that while the constructs share substantial variance, the measures do capture meaningful unique variance for each knowledge type. Because prior studies using the same or similar measures demonstrated clearer discriminant validity in younger students, we interpret the high factor intercorrelations observed here as reflecting knowledge integration rather than as an artefact of poor measurement, consistent with Rittle‐Johnson et al.'s (2001) iterative model's implication that the two knowledge types become increasingly entangled over time. The fact that prior studies with younger samples found discriminant validity under conditions that are likely more favourable (i.e., less integrated knowledge) is consistent with this developmental account, although direct cross‐age comparisons within a single study design would be needed to confirm this. Still, the results highlight that while clear a priori theoretical distinctions between conceptual and procedural knowledge exist (e.g., Byrnes & Wasik, 1991; de Jong & Ferguson‐Hessler, 1996; Hiebert & Lefevre, 1986; Rittle‐Johnson & Schneider, 2015), achieving strong empirical separability through paper‐and‐pencil tests that are intended to assess conceptual and procedural knowledge is challenging.

The results of the first research question hold implications for the design of future measurement instruments. The factor loadings of the four‐factor model indicate which item types most effectively capture each knowledge type. For conceptual fraction knowledge, items assessing magnitude understanding and density understanding were the strongest indicators, outperforming items on part–whole relations. For procedural fraction knowledge, items on fraction addition and subtraction loaded most strongly. For conceptual algebra knowledge, items on equivalence understanding best represented the construct, and for procedural algebra knowledge, items involving equations with parameters were the strongest indicators. These findings can guide researchers and practitioners in selecting or developing items that maximize discriminant power between conceptual and procedural knowledge.

Beyond item selection, future work could explore additional ways to strengthen discriminant validity, for example by using more process‐sensitive measures (e.g., think‐aloud protocols, eye‐tracking, computer‐based assessments capturing response times and solution steps). The items in our study were designed to be solvable through one knowledge type—conceptual items required understanding rather than computation, and procedural items required executing algorithms rather than explaining reasoning—and students' written solution paths gave no indication that they drew on the non‐targeted knowledge type. Nevertheless, as students gain experience, the possibility that they draw on integrated knowledge even for well‐targeted items cannot be fully ruled out with paper‐and‐pencil formats alone.

Investing in such measurement refinements matters because collapsing conceptual and procedural knowledge into a single composite score can obscure theoretically and practically meaningful distinctions. For example, in a follow‐up study, we showed that conceptual (procedural) fraction knowledge longitudinally predicts conceptual (procedural) algebra knowledge, but not procedural (conceptual) algebra knowledge (D'Erchie et al., 2025). Aggregating across knowledge types would have masked these knowledge type‐specific pathways. This highlights that the ability to empirically differentiate between conceptual and procedural knowledge is important for accurately modelling developmental trajectories. More broadly, the ability to empirically distinguish between conceptual and procedural knowledge opens up research directions for understanding students' learning profiles. For example, it allows us to examine why students with similar overall achievement may follow different learning trajectories or respond differently to instruction and enables longitudinal analyses of how each knowledge type develops and interacts with others over time (e.g., Lenz et al., 2024).

### Correlative relationships between conceptual and procedural fraction and algebra knowledge

As the measures captured sufficient unique variance for each construct to warrant separate modelling, our second research question asked how conceptual fraction, procedural fraction, conceptual algebra and procedural algebra knowledge are correlated and whether these correlations differ in strength. The four‐factor model revealed that all six pairwise correlations among the four latent factors were significant and high, ranging from .72 to .88. While prior research established a general link between fraction and algebra knowledge, it had not clarified whether this link is primarily conceptual, procedural or both (e.g., Hurst & Cordes, 2018). Our results show that both conceptual and procedural knowledge are tied across the two domains, and that the correlations are uniformly high. This suggests that the previously observed association between fractions and algebra relies on both knowledge types rather than being driven specifically by conceptual or procedural pathways alone.

However, after correcting for multiple comparisons, none of the 15 pairwise tests comparing the strength of two correlations reached statistical significance (all Bonferroni‐corrected *p*s > .29). Descriptively, however, within‐type cross‐domain correlations tended to be somewhat stronger than cross‐type cross‐domain correlations: conceptual fraction knowledge correlated more strongly with conceptual algebra knowledge (*r* = .86) than with procedural algebra knowledge (*r* = .73), and procedural fraction knowledge correlated more strongly with procedural algebra knowledge (*r* = .82) than with conceptual algebra knowledge (*r* = .77). This descriptive pattern is consistent with the idea that conceptual knowledge across domains draws on similar forms of reasoning (e.g., relational reasoning) and that procedural knowledge across domains relies on shared skills such as familiarity with symbolic notation and algorithmic fluency. However, because none of these differences reached statistical significance, this interpretation is tentative and the data are equally compatible with the possibility that the correlations are genuinely uniform in magnitude, or that the study lacked sufficient power to detect the differences. In a longitudinal follow‐up study, however, we investigated this in more detail and found that students' conceptual fraction knowledge was indeed a stronger predictor of their conceptual algebra knowledge than their procedural fraction knowledge, and likewise their procedural fraction knowledge was a stronger predictor of procedural algebra knowledge than their conceptual fraction knowledge. Moreover, relational and proportional reasoning skills partly mediated the association between conceptual fraction and conceptual algebra knowledge while, contrary to our expectation, we did not find evidence that arithmetic fluency with whole numbers explained associations between procedural fraction and procedural algebra knowledge (D'Erchie et al., 2025).

More broadly, our results contribute to discussions about the relationships between conceptual and procedural knowledge. In this regard, a noteworthy finding concerns the within‐domain correlations (e.g., conceptual fraction with procedural fraction knowledge as well as conceptual algebra with procedural algebra knowledge). In both domains, conceptual and procedural knowledge were highly correlated, especially within algebra (*r* = .88). This finding is consistent with the iterative model. The descriptively stronger coupling in algebra may reflect that conceptual understanding (e.g., understanding equivalence) is directly relevant to procedural success in equation solving.

Lastly, the results do not support the potential intuitive expectation that within‐domain correlations should uniformly exceed cross‐domain correlations. One might assume that conceptual and procedural knowledge within the same domain—say, fractions—would be more strongly correlated than either knowledge type in fractions would be to any knowledge type in algebra. This assumption would make sense because fractions and algebra, after all, are distinct mathematical domains, taught in separate instructional units, at different points in time. However, the present data do not bear this out as we found that procedural fraction knowledge correlated more strongly with conceptual algebra knowledge than with conceptual fraction knowledge. One possible explanation is that the procedural fraction items included tasks such as expanding and simplifying fractions, which require some basic understanding of equivalence, and equivalence understanding was one of the content dimensions along which we operationalized conceptual algebra knowledge.

### Limitations and future directions

A first limitation of our study concerns the internal consistency of the measures for conceptual knowledge of fractions and algebra, respectively, which fell slightly below the commonly accepted threshold of α = .70. However, conceptual knowledge of fractions and algebra are multifaceted constructs. Alpha values can be lower when capturing multifaceted constructs and should not be interpreted as a lack of internal consistency but rather as a reflection of the multifaceted nature of the construct (e.g., Edelsbrunner et al., 2025; Stadler et al., 2021).

Moreover, as briefly discussed above, despite careful item construction based on established conceptualizations, operationalizations and published measures, we cannot guarantee that students exclusively relied on either conceptual or procedural knowledge when solving the items. However, written responses showed no indication of students extensively using conceptual knowledge for procedural items or vice versa. Stronger evidence for the type of knowledge used would require verbal protocols or think‐aloud methods (Faulkenberry, 2013), yet such methods are less feasible in large‐sample studies.

Additionally, the cross‐sectional nature of the data provides only a snapshot of both the measures' discriminant validity and the interrelations among conceptual fraction knowledge, procedural fraction knowledge, conceptual algebra knowledge and procedural algebra knowledge. If conceptual and procedural knowledge become increasingly integrated with instruction, then both the degree of empirical separability and the strength of cross‐domain associations should change over the course of mathematical development. Longitudinal studies tracing the same students across grade levels would be valuable to test whether discriminant validity is indeed stronger at earlier stages and diminishes over time, and how the interplay between conceptual and procedural knowledge within and across domains unfolds developmentally.

Finally, our study focused specifically on fractions and algebra. Future research, however, could explore distinctions and relations between conceptual and procedural knowledge across other mathematical domains, such as geometry, measurement, or functions to better understand whether the results here also generalize across other mathematical domains or whether they are specific to the domains studied here.

## CONCLUSION

The present study showed that paper‐and‐pencil measures of conceptual and procedural knowledge in fractions and algebra show sufficient discriminant validity in experienced students. Yet, all latent factor correlations were relatively high, indicating that empirical separability is achievable but not strong. The results are in line with Rittle‐Johnson et al.'s (2001) iterative model, which implies that conceptual and procedural knowledge should be more integrated in a sample instructed in both domains. Corresponding knowledge types across domains (e.g., conceptual fraction knowledge with conceptual algebra knowledge) tended to be more strongly correlated than non‐corresponding types (e.g., conceptual fraction knowledge with procedural algebra knowledge). Both types of knowledge may contribute to the well‐documented link between fractions and algebra.

## AUTHOR CONTRIBUTIONS

**Michael D'Erchie:** Investigation; writing – original draft; visualization; formal analysis; writing – review and editing. **Claire L. Forsmann:** Investigation; writing – review and editing. **Michael Schneider:** Methodology; writing – review and editing; conceptualization; funding acquisition; project administration; supervision. **Andreas Obersteiner:** Supervision; project administration; methodology; writing – review and editing; conceptualization; funding acquisition.

## FUNDING INFORMATION

This study is part of the *FractAl* project (Fraction Competence and Algebra Competence: Measuring, Modelling, and Predicting Their Inter‐Relations), which was funded by the German Research Foundation (Deutsche Forschungsgemeinschaft (DFG); References: OB 412/4–1 and SCHN 1097/5–1).

## CONFLICT OF INTEREST STATEMENT

All authors declare no conflict of interest.

## Data Availability

The data that support the findings of this study are available from the corresponding author upon reasonable request.

## Footnotes

^1^
The number of items in the algebra test was lower than that of the fractions test, as a pilot study showed that students needed slightly more time to complete the algebra test than the fraction test. In order for students to be comfortably able to solve all items in the time given, prior to the data collection, we decided to reduce the number of items on the algebra scale.

^2^
The list of alternative models is not exhaustive. Other potential models are an item‐level model with the individual items as indicators for the latent variables, or a hierarchical second‐order model with overarching latent factors for fraction and algebra knowledge. These alternative models were tested but failed to converge.
